# Editorial: Signatures of a direct sense of number

**DOI:** 10.3389/fpsyg.2024.1430354

**Published:** 2024-05-29

**Authors:** Wei Liu, Jingguang Li, Yajun Zhao, Xinyu Xie, Guido Marco Cicchini

**Affiliations:** ^1^School of Education, Yunnan Minzu University, Kunming, China; ^2^School of Teacher Education, Dali University, Dali, China; ^3^School of Sociology and Psychology, Southwest University for Nationalities, Chengdu, China; ^4^School of Psychology and Cognitive Science, East China Normal University, Shanghai, China; ^5^Institute of Neuroscience, National Research Council, Pisa, Italy

**Keywords:** numerosity perception, density perception, temporal numerosity, Approximate Number System, magnitude perception, numerosity adaptation, mathematical abilities, math anxiety

Perceiving numerosity, the ability to rapidly estimate the number of discrete elements in a set or presented sequentially, is fundamental to our understanding of the surrounding environment, and serves as an important basis for advanced mathematical thinking. A series of studies have shown interest in how we estimate the number of objects in a scene. However, the mechanisms underlying this are debatable. Some researchers propose a dedicated mechanism for a sense of number (Burr and Ross, 2008), whereas others maintain that numerosity is a second-order product from the combination of continuous magnitudes (Leibovich et al., 2017). To elucidate the debate between the number sense theory and the magnitude sense theory, we delve into the Research Topic with diverse perspectives and methodologies, and present a series of articles exploring the multifaceted signatures of a direct number sense.

The series begins with a study examining the role of area and density in numerosity perception. Previous research suggests that perceived numerosity is biased by area, with larger patches perceived as more numerous (Dakin et al., 2011; Yousif and Keil, 2020). However, the effects of non-numerosity cues are mixed in unmatched patches. To isolate the effects of area, density, and attribute congruence, Liu et al. explored the influence of perceived area inequality, rather than physical area inequality, on numerosity and density perception. Using the Ebbinghaus illusion to induce substantial differences in perceived area, they found no bias in density or numerosity perception when dots were randomly distributed, but a bias toward area in numerosity perception when dots were regularly distributed. This latter distribution can disrupt the numerosity mechanism and facilitate the density mechanism (Liu et al., 2022). These results contradict the idea that numerosity is extracted from the combination of area and density, supporting the view that numerosity is sensed directly. Furthermore, dot number can be inferred by combining density and spatial extent when spontaneous numerosity is disrupted. Previous studies may have overestimated the impact of area inequality on numerosity due to cognitive strategies.

In the temporal domain, stimulus duration and temporal frequency could be similarly exploited to retrieve numerosity. By adapting a psychophysical technique, Cicchini et al. measured the cues that participants used when asked to reproduce a sequence of visual events varying in duration, temporal frequency, and numerosity. Crucially, participants were not asked to reproduce any particular property, which eliminates the potential confounds of cognitive strategies driven by explicit task demands specifying the attributes to be judged (Cicchini et al., 2016, 2019). According to the results, while the overall sequence duration was barely considered, numerosity and temporal frequency were both spontaneously used as the main cues to reproduce the sequences, with a significant dominance of numerosity. This is in line with previous literature that suggests numerosity is directly encoded even for temporal sequences (Arrighi et al., 2014; Togoli and Arrighi, 2021), and indicates that temporal frequency is involved in the numerical process.

The adaptation paradigm provides a powerful tool for studying the number sense. Aulet and Lourenco leveraged this technique to assess whether the visual perception of number is characterized by an opponent or multichannel system. These two systems postulate distinct patterns of adaptation aftereffects observed when participants are adapted to intermediate numerical values. The finding that adaptation leads to repulsive aftereffects aligns with the theory of multichannel coding, where numbers are directly represented, and separate neuronal pools are assigned to each distinguishable numerical value. Examining aftereffects by equally varying test and probe values around adaptor is methodologically sound, and may enhance our understanding of the function of numerosity adaption, for example, how the adaptor serves as a prior for self-calibration (Turi et al., 2015).

A core sense of number, supported by the Approximate Number System (ANS), serves as a basis for mathematical concepts (Izard et al., 2009). A negative correlation exists between ANS performance and math anxiety (MA), but the mechanism remains unclear (Barroso et al., 2021). Mielicki et al. studied whether active control in numerical tasks alters this correlation. Participants either passively viewed or actively chose to view dot arrays and determined the largest quantity. Self-reported MA correlated with accuracy on the passive task, but this correlation disappeared in the active task. This study sheds light on the social-emotional components of our number sense, suggesting that MA leads to anxious ruminations that capture working memory resources necessary for ANS tasks, whereas active control can mitigate this impact.

Overall, this article series highlights the signatures of a direct number sense, providing new insights into its processing mechanisms, neuronal underpinnings, and emotional components. Key findings suggest: (1) numerosity is encoded directly for spatial dot arrays and temporal sequences, rather than recomputed by combining density and area, or sequence frequency and duration; (2) number perception is better characterized by a multichannel system, where numerical values are encoded by separate pools of neurons; (3) anxious ruminations may capture working memory, resulting in a negative correlation between math anxiety and ANS task performance.

## Author contributions

WL: Writing – original draft, Writing – review & editing. JL: Conceptualization, Writing – review & editing. YZ: Conceptualization, Writing – review & editing. XX: Conceptualization, Writing – review & editing. GC: Conceptualization, Supervision, Writing – review & editing.
